# Micro-scale heterogeneity of soil phosphorus depends on soil substrate and depth

**DOI:** 10.1038/s41598-017-03537-8

**Published:** 2017-06-09

**Authors:** Florian Werner, Carsten W. Mueller, Jürgen Thieme, Alessandra Gianoncelli, Camille Rivard, Carmen Höschen, Jörg Prietzel

**Affiliations:** 1 Technical University of Munich, Research Department Ecology and Ecosystem Management, Chair of Soil Science, Emil-Ramann-Straße 2, 85354 Freising, Germany; 2 National Synchrotron Light Source II, Brookhaven National Laboratory, 743 Brookhaven Avenue, Upton, NY 11973-5000 USA; 3 Elettra-Sincrotrone Trieste S.C.p.A., Area Science Park, Basovizza, 34149 Trieste Italy; 4 European Synchrotron Radiation Facility (ESRF), 38000 Grenoble, France

## Abstract

Soils comprise various heterogeneously distributed pools of lithogenic, free organic, occluded, adsorbed, and precipitated phosphorus (P) forms, which differ depending on soil forming factors. Small-scale heterogeneity of element distributions recently has received increased attention in soil science due to its influence on soil functions and soil fertility. We investigated the micro-scale distribution of total P and different specific P binding forms in aggregates taken from a high-P clay-rich soil and a low-P sandy soil by combining advanced spectrometric and spectroscopic techniques to introduce new insights on P accessibility and availability in soils. Here we show that soil substrate and soil depth determine micro-scale P heterogeneity in soil aggregates. In P-rich areas of all investigated soil aggregates, P was predominantly co-located with aluminium and iron oxides and hydroxides, which are known to strongly adsorb P. Clay minerals were co-located with P only to a lesser extent. In the low-P topsoil aggregate, the majority of the P was bound organically. Aluminium and iron phosphate predominated in the quartz-rich low-P subsoil aggregate. Sorbed and mineral P phases determined P speciation in the high-P top- and subsoil, and apatite was only detected in the high-P subsoil aggregate. Our results indicate that micro-scale spatial and chemical heterogeneity of P influences P accessibility and bioavailability.

## Introduction

Phosphorus (P) availability in soils is known to be governed by parent material, the stage of soil development, as well as weathering and erosion intensity^1^. In soils, P limitation often occurs due to unavailability of P^2^. The unavailability is a result of P leaching^1^, strong chemical bonds with other elements such as calcium (Ca), iron (Fe), or aluminium (Al), e.g. as P minerals and as P bound to Al and Fe oxides and hydroxides (oxyhydroxides), as well as to organic matter through metal cations^3^, and immobilization of P in organic residues and microbial biomass^4^. The primary P source in soils, lithogenic apatite, is mobilized during the first 20.000 years of pedogenesis^5^, whereas the relative shares of organically- and soil mineral-bound P species increase with advancing soil development^6^. Micro-scale soil architecture has received increased attention in soil studies^7, 8^ to explain macroscopic soil properties and processes that have been examined for decades^9^. A robust assessment of the chemical and structural accessibility of P requires studying not only bulk P speciation in a soil or soil horizon, but also spatial and chemical P heterogeneity at the micro-scale. At the moment, only scant information^10^ exists on spatial soil P micro-distribution patterns due to the lack of highly versatile, affordable analytical methods, instruments, and standardized data analysis^11^. Established chemical fractionation techniques of bulk soil^12^, and also advanced techniques of P speciation, as e.g. solution 31 P nuclear magnetic resonance spectroscopy^13^ are inappropriate, because they destroy the structure of soil aggregates.

Nanoscale Secondary Ion Mass spectrometry (NanoSIMS) enables high-resolution element distribution mapping while preserving the overall structural integrity, however eroding a sample surface at a nm scale^14^. Moreover, improvements of synchrotron-based X-Ray Fluorescence (μ-XRF) spectroscopy and mapping allow *in-situ* assessments of element distributions at the micro-scale^10^. In our study, both techniques were combined to overcome individual technique limitations, such as matrix effects for NanoSIMS^14^ and the comparably large beam penetration depth for μ-XRF spectroscopy^15^. Additionally, direct P speciation of micro-environments can be obtained by synchrotron-based X-Ray Absorption Near Edge Structure (μ-XANES) spectroscopy^16, 17^. Combining these techniques thus provides powerful data on soil P distribution and speciation at the micro-scale and allows a *direct* assessment of P accessibility in soil systems^18^. We hypothesise that in soil aggregates, chemical and structural P accessibility at the micro-scale is determined by the distribution of total P and different P species. Here, we show that micro-scale spatial and chemical P heterogeneity is related to soil depth and soil substrate in four undisturbed aggregates of different horizons from two forest soils, which have developed from siliceous parent material with low and high-P content, respectively. Information is provided on the micro-scale P distribution as related to the distribution of major soil compounds, such as pedogenic soil minerals and soil organic matter (SOM), and on major P binding forms at 15 P micro-sites (three to four sites per sample) using, for the first time, a combination of spatially-resolving techniques, and an original data analysis procedure (details: see Methods section). Our research opens new perspectives on how P accessibility and thus availability in soils is influenced by the micro-scale spatial and chemical heterogeneity of soil P.

## Results

### Patterns of micro-scale soil P distribution

**Figure 1:**
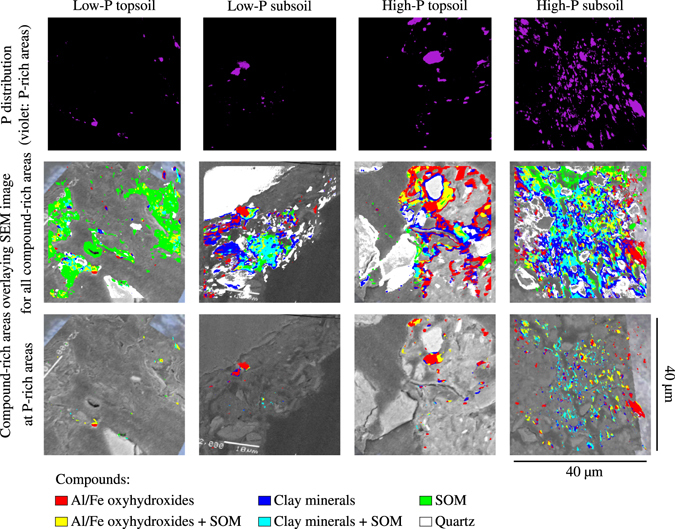
Clustered Nanoscale Secondary Ion Mass spectrometry (NanoSIMS) images of topsoil and subsoil micro-sites in aggregates of two forest soils. Top: Distribution maps of phosphorus (P)-rich areas after clustering (definition: see Methods section). Middle: All compound-rich areas overlaying scanning electron microscope (SEM) images. Bottom: Only those compound-rich areas that co-locate with P at the P-rich areas, overlaying SEM images.

Raster imaging performed with NanoSIMS revealed that the micro-spatial P distribution was characterised by pronounced heterogeneity (Fig. 1, upper section). The relatively few P-rich areas (definition: see Methods section and Fig. S1) in the aggregates of the low-P soil showed larger distances from each other than the relatively abundant P-rich areas in the aggregates of the high-P soil. Phosphorus-rich areas were predominantly co-located with Al and Fe (mostly oxyhydroxides) and to a lesser extent with clay minerals (Fig. 1, lower section).

In the aggregates of the low-P soil, more P-rich areas were co-located with Al/Fe oxyhydroxides in the subsoil (59–85%) than in the topsoil (44–62%, Table 1), but topsoil Al/Fe oxyhydroxides were more frequently co-located with P than subsoil Al/Fe oxyhydroxides (Table S1). Additionally, SOM was strongly co-located with P in the low-P topsoil (16–31%, Table 1).

**Table 1:** Phosphorus (P)-rich areas that are co-located with aluminium and iron (Al/Fe) oxyhydroxides, clay minerals, quartz, and soil organic matter (SOM).

		P-rich areas that are co-located with				
		Al/Fe oxy-hydroxides^*a*^	clay minerals^a^ (% of total P-rich area)	quartz	SOM	unspec-ified
low-P topsoil	ELETTRA	49 (41)	2 (2)	—	31	18
	ESRF	62	14	1	—	24
	NanoSIMS1	61 (48)	16 (16)	1	19	3
	NanoSIMS2	44 (37)	21 (18)	7	16	12
low-P subsoil	ELETTRA	85 (37)	8 (6)	1	3	4
	ESRF	59	25	5	—	11
	NanoSIMS	70 (7)	30 (10)	—	—	—
high-P topsoil	ELETTRA	89 (43)	2 (2)	—	3	6
	ESRF	64	30	3	—	2
	NanoSIMS1	50 (45)	41 (35)	1	5	4
	NanoSIMS2	81 (22)	17 (3)	—	—	1
high-P subsoil	ELETTRA	89 (17)	3 (3)	—	1	8
	ESRF	91	4	2	—	3
	NanoSIMS1	59 (37)	34 (24)	1	3	4
	NanoSIMS2	49 (25)	48 (31)	1	1	2

^*a*^Brackets: share of compound that is also co-located with SOM.

Nanoscale Secondary Ion Mass Spectrometry (NanoSIMS) and synchrotron-based X-Ray Fluorescence (μ-XRF) mapping (at the synchrotrons ELETTRA, Italy, and ESRF, France) were used. In three aggregates, μ-XRF imaging included two NanoSIMS measurements (displayed as subscript 1 and 2).

In the high-P aggregate, Al and Fe oxyhydroxides were co-located with P-rich areas in high percentages in both top- (50–89%) and subsoil (49–91%, Table 1). Additionally, Al/Fe oxyhydroxides and clay minerals were frequently co-located with P in this soil, especially in the topsoil (Table S1).

### Aggregate P speciation at the micro-scale

**Figure 2:**
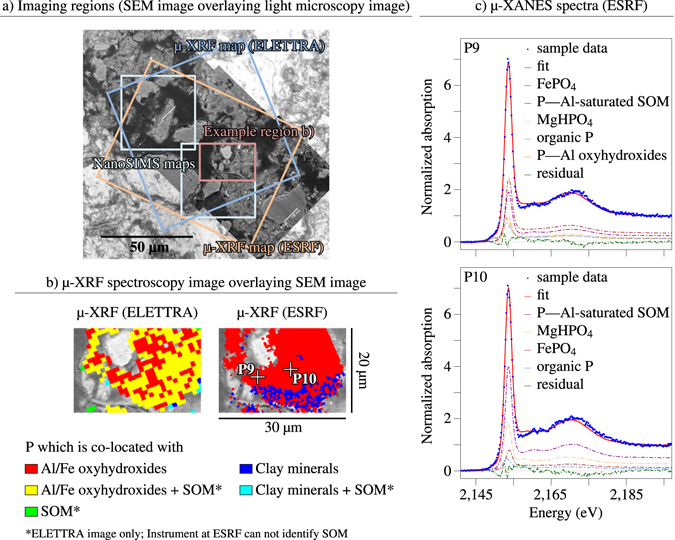
Example of micro-scale X-Ray Fluorescence (μ-XRF) and X-Ray Absorption Near Edge Structure (μ-XANES) spectroscopy. (**a**) Scanning electron microscope (SEM) (back scattered electron) images overlaying light microscope image of the soil aggregate from the high-Phosphorus (P) topsoil, raster imaging regions of Nanoscale Secondary Ion Mass spectrometry (NanoSIMS) and μ-XRF mapping, indicated as rectangles, (**b**) exemplary detail of μ-XRF mapping results obtained at ELETTRA and ESRF from P-rich areas, (**c**) two μ-XANES spectra from selected P micro-sites (P9 and P10, ESRF) with the linear combination fit, and calculated shares of P species.

Spatially-resolved P speciation results using μ-XANES spectroscopy revealed that P micro-sites (definition: see Methods section, fitted spectra: see Figs. 2c﻿ and S2) in the low-P topsoil aggregate mostly comprised organic P (0–55% of total P: free organic P, 9–38% calcium (Ca)-bound organic P, Table 2). Moreover, we identified considerable amounts of orthophosphate adsorbed to Al oxyhydroxides (27–45% of total P, Table 2) and of organic P adsorbed to Fe oxyhydroxides (0–43%, Table 2). All soil compounds were to a larger extent co-located with P in the topsoil than in the subsoil (Table S1).

**Table 2:** Results from linear combination fitting of synchrotron-based X-Ray Absorption Near Edge Structure (XANES) spectra by various phosphorus (P) reference standards (details: see Methods section).

		Organic P	Ca-bound organic P	Apatite	MgHPO4	AlPO4^*a*^	FePO4^*a*^	organic P adsorbed to	orthophosphate adsorbed to				
								Fe oxyhy-droxides (% of total P)	Fe oxyhy-droxides	Al oxyhy- droxides	Al-satura-ted clays	Al-satura-ted SOM	R-factor
low-P topsoil	P1	—	36	—	—	—	—	21	—	43	—	—	0.0047
	P2	16	9	—	—	—	—	43	—	32	—	—	0.0029
	P3	9	38	—	—	—	—	8	—	45	—	—	0.0034
	P4	55	17	—	—	—	—	—	—	27	—	—	0.0040
low-P subsoil	P5	—	—	—	—	8	61	14	—	—	17	—	0.0032
	P6	—	—	—	—	32	48	20	—	—	—	—	0.0026
	P7	—	—	—	—	100	—	—	—	—	—	—	0.0037
high-P topsoil	P8	—	—	—	—	37	16	5	8	26	—	7	0.0012
	P9	13	—	—	16	—	34	—	—	12	—	24	0.0019
	P10	9	—	—	28	—	13	—	—	—	—	51	0.0028
	P11	—	—	—	19	45	9	—	—	13	—	14	0.0037
high-P subsoil	P12	—	—	35	—	51	8	—	—	6	—	—	0.0014
	P13	—	—	46	—	46	—	—	—	9	—	—	0.0025
	P14	—	10	17	—	—	28	6	—	40	—	—	0.0045
	P15	—	—	7	—	35	—	—	11	33	14	—	0.0014

^*a*^Includes orthophosphate bound to Al and Fe oxyhydroxides by surface precipitation, respectively.

Fifteen P micro-sites (P1 to P15) were investigated on soil aggregates. Quality of the fit given by R-factor; accuracy of the fitting 5–10%^36^.

Scanning electron microscope (SEM) imaging revealed that the aggregates sampled in the low-P subsoil consisted of quartz grains which were encompassed with and agglutinated by a finer-grained matrix (Fig. S3). Phosphorus was incorporated in the fine matrix of mineral and SOM coatings (Fig. 1), and the retained P was predominantly inorganic (Tables 1 and 2). In the low-P subsoil aggregate, spatially-resolved P speciation conducted on P micro-sites showed that P was predominantly bound as AlPO4 (8–100% of total P) and FePO4 (0–61%, Table 2). In addition to Al/Fe phosphate-bound P, up to about a quarter of total P in the low-P subsoil aggregate was adsorbed to Al-saturated clay minerals and/or Fe oxyhydroxides (Tables 1 and 2).

As expected, P-rich micro-sites in the high-P topsoil aggregate were dominated by orthophosphate adsorbed to Al-saturated SOM (7–51% of total P) and Al oxyhydroxides (0–26%), as well as by AlPO4 (0–45%) and FePO4 (9–34%, Table 2). Fe oxyhydroxides influenced P binding marginally in the high-P topsoil aggregate (Table 2). Phosphorus bound in organic compounds was only a minor P species in this aggregate (0–13%, Table 2). Unexpectedly, MgHPO4 was detected in the high-P topsoil aggregate (0–28%, Table 2). In contrast, apatite was detected solely, but consistently in the aggregate of the high-P subsoil (7–46%, Table 2). Similar to topsoil percentages, we found 0–51% of AlPO4 and 0–28% of FePO4 (Table 2). Phosphorus adsorbed to Al-saturated clay minerals contributed only minor to P speciation in the high-P subsoil aggregate (0–14%, Table 2).

## Discussion

### Micro-scale soil P distribution patterns are soil-dependent

The investigated aggregates showed striking differences with respect to the amount, distribution, and form of P-rich areas. At P-rich areas of the low-P topsoil aggregate, SOM was strongly co-located with P (Table 1), and co-localisation of P with Al/Fe oxyhydroxides and clay minerals increased when SOM was present (Table S1). In accordance, P was mainly bound organically in the bulk soil of the low-P topsoil (Table S2). These results show that in the sandy, quartz-rich low-P topsoil, the few Al and Fe oxyhydroxides and clay minerals, i.e. smectite and illite (Table S3) play a pivotal role in retaining P. The co-localisation of these compounds with SOM indicates that P-rich SOM is probably bound in complexes at the compound surfaces^19^.

The aggregates of the high-P soil showed different P distribution patterns compared to the aggregates of the low-P soil. Here, Al and Fe oxyhydroxides were the dominant partners for co-localisation with P (Table 1). About half of the total P was bound organically in the bulk soil of both top- and subsoil (Table S2). Bulk soil studies have long pointed out that the large surface areas of Al and Fe oxyhydroxides and Al-saturated high-activity clay minerals provide numerous sites to strongly bind inorganic and organic soil P, especially under acidic conditions^19^, as present at this site (Table S2). The influence of clay minerals on P retention is exerted mostly by P sorption on cationic Al hydroxy polymer clusters on 2:1 clay mineral surfaces^20, 21^. In both depths at the high-P site, illite and chlorite were the main phyllosilicates in the clay fraction (Table S3). Thus, apart from the presence and ion occupancy of P binding partners, the structural and chemical P accessibility in soil aggregates also depends on the heterogeneous distribution of P and different P species at the micro-scale. This implies that studies aiming at a robust mechanistic understanding of micro-scale P retention and P release as well as P accessibility and availability for plants and soil micro-organisms must account for the heterogeneous P distribution in soil aggregates.

### Soil depth considerably influences P heterogeneity at advanced stage of pedogenesis

The soils on both sites were pronouncedly acidified (Table S2), but only the sandy low-P soil exhibited podzolization. Phosphorus distribution and micro-site P speciation differed strongly between the low-P top- and subsoil, whereas this was not the case for the high-P top- and subsoil. Our findings from the aggregates of the low-P soil indicate that soil depth is a major determinant of P speciation on the micro-scale, particularly at later stages of pedogenesis. In the low-P topsoil aggregate, P species were mainly organic (Table 2). Due to the high Ca ion content (Table S2), we suppose that the soil has been limed in the past, supporting the formation of Ca-P complexes^22^ in the topsoil. Organically-bound P is subject to mineralisation^4^ and thus generally accessible to plants and microbes. However, we suppose that the few Al and Fe oxyhydroxide-bound P resources are structurally inaccessible due to occlusion.

In the subsoil aggregate of the low-P aggregate, P was especially scarce (Table S2). The fine matrix of mineral and SOM coatings surrounding larger quartz grains (Fig. S3) contained mostly phosphate minerals (Tables 1 and 2). Even though there are doubts that Fe and Al phosphates, such as strengite or variscite can persist in soil environments with moderately acidic pH^23^, crystalline AlPO4 (berlinite) was found to be more resistant against dissolution at pH 6–7 than at pH 3^24^. In this context it must be emphasized that the XANES spectrum of amorphous AlPO4 is identical with that of Al hydroxy phosphate, which is thermodynamically stable at moderately acidic pH values. In addition, the presence of Al hydroxy phosphate is more likely due to the large amount of secondary, primarily Al(OH)3-, chlorite in the subsoil, but not in the topsoil clay fraction (Table S3). Even though Al and Fe are highly important for retaining P in sandy subsoil aggregates, the scarce P resources in these aggregates are presumably chemically, as well as structurally inaccessible to plants and microbes.

### Soil substrate influence on P heterogeneity is stronger during early stages of pedogenesis

In aggregates of the high-P soil, P speciation and distribution patterns are governed by the clayey-silty soil substrate, which has originated from ongoing intense weathering of basalt rock fragments into Al and Fe oxyhydroxides and 2:1 clay minerals^25^. For example, the top- and subsoil percentages of AlPO4, FePO4, and of orthophosphate adsorbed to Al or Fe oxyhydroxides (Table 2) are similar, which is not the case for the pedogenetically older low-P soil. In contrast to our μ-XANES results on P micro-sites, no AlPO4 had been identified for bulk soil samples at the high-P site (30–70 cm depth) in a recent XANES spectroscopy study^26^. Bulk soil XANES spectra are dominated by a diffuse background of low-P content^18^ and therefore often fail to represent P micro-sites in the interior of soil aggregates. Thus, we surmise that even though considerable AlPO4 (Al hydroxy phosphate) is bound at P micro-sites, this P form is below detection limit in bulk soil samples. Speciation results using μ-XANES spectroscopy of a transect from the agglutinating soil matrix to the interior of an Al-Fe concretion in the high-P subsoil (Fig. S4) also show that the proportion of AlPO4 was increased especially in the matrix, whereas sorbed P species dominated at the concretion surface (Fig. S4).

Most noticeable, we expected higher percentages of clay mineral-bound P in the high-P aggregates. At a pH value of about 4, which was present in the high-P subsoil (Table S2), negatively charged surfaces of expandable clay minerals are mostly covered with Al hydroxy cations and thus have a P sorption efficiency similar to that of Al oxyhydroxides^21^. About two-thirds of the bulk soil mass was silt sized particles which were dominated by quartz and augite and no expandable clay minerals were detected (Table S3). In the high-P topsoil aggregate, SOM more pronouncedly influenced Al exchanger cations than clay minerals (Table 2). We assume that in the high-P topsoil, P remains accessible due to ongoing detachment of P after mineralisation of SOM. However, P also leaches as dissolved organic P and/or in colloids through the soil, as described for a similar German forest soil site^27^. In the subsoil, apatite weathering provides accessible P resources, even though other P species are structurally inaccessible. Thus, our results emphasize not only the importance of sorbed and mineral P phases at early stages of pedogenesis, but also the importance of investigating also micro-scale P heterogeneity when assessing P accessibility in soils.

### Micro-scale P speciation patterns support micro-reactor concept

The “micro “chemical reactor concept” states that each soil micro-site represents an independent micro-reactor of unique chemical composition^8^. Even though individual reactors are interconnected with each other and adjacent compounds, each analysis is regarded as a separate perspective on a complex system. The presence of AlPO4 and Al hydroxy phosphate in the high-P soil is an example where the chemical composition at P micro-sites enables understanding the characteristics of the chemical transformations that must have occurred within these micro-sites. Another example is the existence of MgHPO4 in our high-P topsoil sample (Table 2). This is surprising, as this compound is not stable in acidic solutions below pH 5. However, in contrast to the low-P soil, in the P-rich soil primary, i.e. Mg(OH)3-chlorite made up 20–30% of the total clay mineral content (Table S3). Thus, our XANES spectroscopy and XRD data suggest that, similar to clay-bound Al(OH)3 in the P-poor soil, as reported above, clay-bound Mg(OH)3, probably occluded in less acidic interior regions of soil aggregates, is a major P-bearing phase in the high-P soil. The same occlusion can be stated for orthophosphate adsorbed by Al oxyhydroxides in the high-P and low-P topsoil aggregate (Table 2). These results indicate that some biocycled P is retained at micro-sites in the topsoil aggregates of both sites by pedogenic Al oxyhydroxides due to occlusion, which furthermore supports the micro-reactor concept.

## Conclusions

Micro-scale P distribution and speciation patterns were investigated in soil aggregates to increase our knowledge about P accessibility in soils. We demonstrated for the first time that Al/Fe oxyhydroxides and clay minerals are major binding partners of P in such aggregates. Our results showed that P species diversity and micro-scale distribution predominantly is dependent on soil substrate and soil depth. We showed that the analysis of P speciation at micro-sites is relevant for understanding the chemical changes in a soil micro-chemical reactor^8^. Four factors governing P availability are well-known: i) parent material, ii) stage of pedogenesis, iii) weathering intensity, and iv) erosion (as P input)^1^. Our study showed that the use of spatially-resolving tools is necessary to understand the governing factors of P availability, as related to the micro-scale spatial and chemical heterogeneity of soil P. The benefit of our study is not only based on effectively combining distribution and speciation analysis for soil aggregate studies, but also on opening these new perspectives on P availability in soils.

## Methods

### Soil material and sample preparation

Soil samples were obtained from two sites that were stocked with mature *Fagus sylvatica* forests of about 120 years of age, located in Germany, near Lüß (LU), Gauss-Krüger-coordinates: 3585473 E, 5857057 N, and Bad Brückenau (BB), Gauss-Krüger-coordinates: 3566195 E, 5579975 N. The sites are part of the International Co-operative Programme on Assessment and Monitoring of Air Pollution Effects on Forests (ICP Level II). They differed in soil and beech leaf P contents, and were end-members of a forest P availability gradient^28^. The soils were formed from siliceous parent material under temperate climate and were classified^29^ as Hyperdystric Folic Cambisol (LU), and as Dystric Skeletic Cambisol (BB). The LU soil has formed from Pleistocene glacifluvial sands, the BB soil from basalt. A basic soil characterisation can be found in Table S2. In the main text, the sites LU and BB are named as low- and high-P sites, respectively.

Samples were taken from the mineral topsoil (directly below the organic layer) and the subsoil (30 cm depth) with a steel tube (∅2 cm, sampling depth 3 cm). They were dried at 60 °C for 48 hours and subsequently sieved (<2 mm). For each site and depth, three dried, intact soil aggregates (size approximately 1–2 mm^3^) were selected randomly from the dried bulk soils and embedded in an epoxy resin (Araldite 502 Kit, Electron Microscopy Sciences, Hatfield, PA, USA). The embedded aggregates were then cured at 60 °C for 24 hours, and subsequently thin-sectioned, polished and coated with gold by physical vapor deposition^30^. One aggregate per site was randomly selected for scanning electron microscopy (Jeol JSM 5900LV, Eching, Germany) imaging to locate regions of interest for the subsequent imaging techniques.

### Assessing element distributions using NanoSIMS

The NanoSIMS measurements were conducted with a Cameca NanoSIMS 50 L instrument (Cameca, Gennevilliers Cedex, France) at Technical University of Munich, Germany. A Cs^+^ source with a primary ion energy of 16 keV was used to produce secondary ions of the sample surface. The focused beam (lateral resolution about 100 nm) scanned over areas of 40·40 μm^2^ while the mass signals of the secondary ions ^12^C-, ^16^O-, ^12^C^14^N-, ^28^Si-, ^27^Al^16^O-, ^31^P^16^O^2^-, and ^56^Fe^16^O- (C, O, CN, Si, Al, P, Fe) were collected. In the latter three ions ionization is stronger than that of the individual Al, P, or Fe ions. The ion images were acquired using a 10 ms/pixel dwell time in an area of 512·512 pixels^2^. On each aggregate 5–7 measurements were conducted.

### Assessing element distributions using μ-XRF

We performed μ-XRF at the same sample locations as the NanoSIMS measurements (Fig. 2a). However, due to beam time limitation restrictions for measuring at the synchrotrons not all micro-regions analysed by NanoSIMS measurements could be evaluated with μ-XRF. Synchrotron-based μ-XRF measurements were conducted at the TwinMic Beamline of the ELETTRA synchrotron (storage ring energy 2.0 GeV) in Trieste, Italy, and at Beamline ID21 of the European Synchrotron Radiation Facility (ESRF, storage ring energy: 6.03 GeV) in Grenoble, France. The TwinMic Beamline was operated in low energy X-Ray Fluorescence (LEXRF) mode and was equipped with a 600 lines per mm Au plane-grating monochromator. The fluorescence detector consisted of 8 silicon drift detectors (SDD)^31^. The samples were installed in a vertical plane, orthogonally to the incident photon beam. The data was acquired at 2.19 keV to optimize the P emission signal. The LEXRF dwell time varied between 1 and 7 s as a function of the samples, and the maps were acquired by raster scanning with a 1 μm step size and a minimum size of 40·40 pixels^2^ (=40·40 μm^2^).

The ID21 Beamline^32^ of ESRF was equipped with a double crystal Si(111) monochromator (energy resolution: 0.4 eV). The samples were tilted by 28 with respect to the incident beam, and the fluorescence signal was collected by a SDD detector, placed at a 49 angle with respect to sample surface. Microμ-XRF maps were obtained by raster scanning using a focused beam. After selection of the area of interest, maps were recorded at 2.165 keV to intensify P *K*-edge emission, with a dwell time of 150 ms and a step size of 0.5 μm. The elemental distributions were obtained by deconvoluting the μ-XRF spectra after incoming flux (and detector deadtime) correction, on maps of minimum size of 80·80 pixels^2^ (40·40 µm^2^) with the PyMCA software^33^.

### Co-localisation analysis for determining distribution patterns

All XRF and NanoSIMS measurements were analysed with the statistical software *R*, Version 3.3.1^34^. First, we performed k-means cluster analysis^11^ (Fig. S1) on every ion/element count rate of an ion/element to determine regions with similar element identity. The count rates of every pixel were assigned to one of five cluster centres, respectively. The three largest cluster centre values were combined to result images where each pixel is either assigned as “area rich in”, or as a negligible count value (Fig. S1). For the NanoSIMS images, P, Fe, Al, CN and Si were selected. These latter four ions and their combinations were then assigned to soil compound classes: (i) Fe/Al oxyhydroxides (Al, Fe, Fe + Al; also including Al and Fe oxyhydroxide surface precipitates), (ii) Fe/Al oxyhydroxides + SOM (Al + CN, Fe + CN, Fe + Al + CN, Fe + CN + Si), (iii) clay minerals (Al + Si, Fe + Al + Si), (iv) clay minerals + SOM (Al + CN + Si, Fe + Al + CN + Si), (v) quartz (Si), (vi) SOM (CN, CN + Si), and (vii) unspecified. The total number of pixels, respective total area, of these compounds and the compound-rich areas that were co-located with P were counted (Fig. 1). Dividing the P-rich area that is co-located with a specific compound by the total P-rich area resulted in a proportional measure for P binding. Dividing the compound-rich area that is co-located with P by the total compound-rich area resulted in a proportional measure for compound P allocation. Both measures are displayed as percentage of total P/compound-rich area, respectively.

μ-XRF maps were processed alike, but the elements for co-localisation differed due to different instrument conditions (Fig. 2b). The ELETTRA instrument also allowed all stated P binding categories, however, CN was replaced by N only. The attribution to the seven categories persisted as stated. The ESRF instrument unfortunately did not provide information on N, but on the *L2*-edge of Fe (719.9 eV). During compound classification, this element was treated similarly as the first Fe edge. The compound classes, to which element combinations were assigned to, were therefore limited to only those that did not include SOM (i, iii, v, and vii). This procedure and the heterogeneity of P and soil compounds resulted in different proportions of P and soil compounds depending on the instrument used.

### Assessing P speciation using μ-XANES spectroscopy and spectra fitting

μ-XANES measurements were conducted at Beamline ID21 of ESRF to support the elemental raster images by direct P speciation results from linear combination fitting (LCF) with P reference spectra of all relevant P species (Fig. 2b). Phosphorus *K*-edge μ-XANES spectra were collected with a 0.2 eV step size, a dwell time of 0.1 s, and in an energy range from 2.13 to 2.20 keV. For each P micro-site (minute sites of increased P content), 10 to 40 spectra were recorded and merged. For LCF, we used 17 P standards, which represent P species in temperate forest soils, and whose *K*-edge XANES spectra had been acquired at beamline 8 of the Synchrotron Light Research Institute (SLRI) in Nakhon Ratchasima, Thailand:^35^ (1) crystalline and (2) amorphous FeIIIPO4 (its proportions combined, termed as FePO4), (3) crystalline and (4) amorphous AlPO4 (its proportions combined, termed as AlPO4), (5) phytic acid sodium salt (IHP, termed as organic P), (6) apatite, (7) MgHPO4, (8) orthophosphate and (9) IHP retained by boehmite (termed as orthophosphate and organic P adsorbed to Al oxyhydroxides), (10) orthophosphate and (11) IHP retained by ferrihydrite (termed as orthophosphate and organic P adsorbed to Fe oxyhydroxides), (12) orthophosphate and (13) IHP retained by Al-saturated montmorillonite (termed as orthophosphate and organic P adsorbed to clay minerals), (14) orthophosphate and (15) IHP retained by Al-saturated soil organic matter (termed as orthophosphate and organic P adsorbed to SOM), P retained by (16) precipitated Ca3-IHP (termed as Ca-bound organic P), (17) IHP adsorbed to CaCO3^21^. Spectra were calibrated in energy by comparing apatite spectra taken at SLRI and ESRF, a correction value of *δ* E = −1.15 eV was applied.

μ-XANES spectra were base-line corrected and edge-step normalized following a standard protocol published recently^36^ using the statistical software *R*, Version 3.3.1^34^. The reference spectra were base-line corrected from −36 to −15 eV and normalized from +37 to +57 eV with respect to the edge-step of the respective spectra. As for the samples, the first base-line correction parameter was allowed to vary from −28 to −18 eV (step: 1 eV) and the second from −16 to −8 eV (step: 0.5 eV) with respect to the edge-step. The first normalization parameter was allowed to vary between +29 and +39 eV (step: 0.5 eV) and the second between +42 and +48 eV (step: 1 eV) with respect to the edge-step. The actual LCF was performed from −14 to 46 eV with respect to the edge-step of a sample spectrum. Phosphorus speciation shares below 5% of total P were excluded and LCF was repeated without the respective standards. Only fits with R-factors smaller than 0.005 were included because fits with R-factors greater than this value were obviously unreliable. Results from LCF (Figs. 2c and S2) only show those P reference spectra proportions that were detected as more than 5% of total P at least once.

### Data availability

The Supplementary Tables and Figures are provided on the *Nature Scientic Reports* website at doi:nat/scirep.doi. Raw NanoSIMS and μ-XRF mapping data (including cluster center assignment), as well as raw μ-XANES spectra are available on the public data repository website of *PANGAEA* at doi:10.1594/PANGAEA.874444. The *R* code for linear combination fitting of P *K*-edge XANES spectra has been published at the *Comprehensive R Archive Network* under the package name *LCF*.

## Electronic supplementary material

Supplementary Information
