# Supplementary material for: Micro-scale heterogeneity of soil phosphorus depends on soil substrate and depth
Source: Sci Rep. 2017 Jun 9;7:3203. doi: 10.1038/s41598-017-03537-8 (PMC5466645; doi:10.1038/s41598-017-03537-8)
Supplement: Supplementary file 1 — Supplementary Information [file 41598_2017_3537_MOESM1_ESM.pdf]

SUPPORTING INFORMATION

for

Micro-scale heterogeneity of soil phosphorus depends on soil substrate and depth

by

Florian Werner[1,*], Carsten W. Mueller[1], Jürgen Thieme[2], Alessandra Gianoncelli[3], Camille Rivard[4], Carmen Höschen[1] & Jörg Prietzel[1]

[1]Technical University of Munich, Research Department Ecology and Ecosystem Management, Chair of Soil Science, Emil-Ramann-Straße 2, 85354 Freising, Germany

[2]National Synchrotron Light Source II, Brookhaven National Laboratory, 743 Brookhaven Avenue, Upton, NY 11973-5000, USA

[3]Elettra-Sincrotrone Trieste S.C.p.A., Area Science Park, Basovizza 34149, Trieste, Italy

[4]European Synchrotron Radiation Facility (ESRF), 38000 Grenoble, France

[*]corresponding author: florian.werner@wzw.tum.de

Number of pages: 9
Number of tables: 3
Number of figures: 4

Table S1. Compound-rich areas that are co-located with phosphorus (P) as percentage to all compound-rich areas of aluminium and iron (Al/Fe) oxyhydroxides, clay minerals, quartz, and soil organic matter (SOM). Nanoscale Secondary Ion Mass Spectrometry (NanoSIMS) and synchrotron-based X-Ray Fluorescence (μ-XRF) spectroscopy (at the synchrotrons ELETTRA, Italy, and ESRF, France) were used. In three aggregates, μ-XRF spectroscopy imaging included two NanoSIMS measurements (displayed as subscript 1 and 2).

		Area enriched in compound (co-located with P)						
		Al/Fe oxy-hydroxides		clay minerals		quartz	SOM	unspec-ified
		only	+SOM[a]	only	+SOM[a]			
		(% of total compound-rich area)						
low-P topsoil	ELETTRA	37	39	–	48	–	30	6
	ESRF	54	–	41	–	3	–	15
	$NanoSIMS_1$	13	10	–	5	–	1	–
	$NanoSIMS_2$	3	4	2	8	1	1	–
low-P subsoil	ELETTRA	18	21	12	10	1	3	4
	ESRF	36	–	23	–	2	–	10
	NanoSIMS	17	4	2	2	–	–	–
high-P topsoil	ELETTRA	85	60	17	11	4	4	7
	ESRF	73	–	55	–	3	–	3
	$NanoSIMS_1$	28	46	10	43	–	1	–
	$NanoSIMS_2$	15	18	6	6	–	–	–
high-P subsoil	ELETTRA	26	63	–	27	–	5	8
	ESRF	32	–	3	–	2	–	13
	$NanoSIMS_1$	33	47	19	46	1	3	1
	$NanoSIMS_2$	36	36	11	26	–	1	1

[a]Share of compound that is also co-located with SOM

Table S2. Basic soil characterisation of the study sites and depths. Sites and depths are characterised by soil texture, pH[a], organic carbon (C_{org})[b], organic phosphorus (P_{org})[c], total phosphorus, calcium, magnesium, potassium, sodium, aluminium, iron (P_{tot}, Ca_{tot}, Mg_{tot}, K_{tot}, Na_{tot}, Al_{tot}, Fe_{tot})[d], dithionite-citrate-bicarbonate extractable P, Al, Fe (P_{dcb}, Al_{dcb}, Fe_{dcb})[e], oxalate extractable P, Al, Fe (P_{ox}, Al_{ox}, Fe_{ox})[f], effective cation exchange capacity (CEC_{eff})[a], cations (Ca, Mg, Na, K, Al, Fe, manganese [Mn], Hydrogen [H])[a], and base saturation (BS)[a].

	C_{org}	P_{org}	P_{tot}	Ca_{tot}	Mg_{tot}	K_{tot}	Na_{tot}	Al_{tot} (mg g^{-1})	Fe_{tot}	P_{dcb}	Al_{dcb}	Fe_{dcb}	P_{ox}	Al_{ox}	Fe_{ox}
low-P topsoil	232.08	0.23	0.22	0.98	0.42	3.64	1.52	8.50	4.98	0.17	1.07	2.91	0.11	1.15	1.30
low-P subsoil	6.22	0.03	0.06	1.38	0.45	6.28	1.80	11.44	6.01	0.03	0.71	3.51	0.01	0.68	1.27
high-P topsoil	97.47	1.15	3.12	6.80	18.04	8.73	4.81	51.31	74.90	2.59	8.27	40.50	2.19	7.21	21.51
high-P subsoil	35.85	1.04	1.99	15.94	42.63	8.51	4.52	61.67	87.81	1.18	7.70	37.14	1.13	8.11	14.30

	soil texture	pH ($CaCl_2$)	CEC_{eff}	Ca	Mg	Na (mmolc kg^{-1})	K	Al	Fe	Mn	H	BS (%)
low-P topsoil	loamy sand	3.0	35.1	2.8	1.0	0.6	0.7	11.1	1.2	0.2	17.4	15
low-P subsoil	sand	4.3	19.0	1.0	0.2	0.4	0.5	16.5	0.3	0.1	0.0	11
high-P topsoil	silty clay loam	3.2	142.9	30.2	13.9	0.5	4.8	70.2	12.2	1.8	9.4	35
high-P subsoil	loam	4.0	77.1	8.2	5.3	0.3	1.6	57.6	0.3	3.2	0.7	20

[a]pH, CEC_{eff}, cations, and BS values from low-P topsoil (0 – 5 cm), low-P subsoil (20 – 40 cm) [low-P soil: Meesenburg, pers. comm.], high-P topsoil (0 – 6 cm), high-P subsoil (6 – 38 cm) [high-P soil: Dietrich, pers. comm.]

[b]Analysed with Elementar VarioEL CN analyzer (Elementar GmbH, Hanau, Germany)

[c]Determined using ignition loss method[1]

[d]Obtained by total digestion of bulk soil with $HF/HClO_4$ and subsequent analysis using inductively coupled plasma optical emission spectrometry (Vista-PRO Simultaneous ICP-OES, Varian Inc., Palo Alto, CA, USA)

[e]Extraction using bicarbonate-buffered dithionite-citrate solution[2,3]

[f]Extraction using acidic NH_4 oxalate[4]

Table S3. Mineral classification in soil fractions. The fractionation was achieved with sieving and Atterberg cylinders. Different mineral types were identified by X-ray diffractometry (Philips PW 1830, X-ray source: Co *K* alpha).

		**low-P topsoil**			**low-P subsoil**		
		sand	silt	clay	sand	silt	clay
		(weight-% of total bulk soil mass)					
		84	14	2	86	13	2
		sand	silt	clay	sand	silt	clay
		(weight-% of fraction mass)					
minerals	quartz	90	70 – 80	30 – 40	95	80	30
	K and Ca feldspars	10	20 – 30	10 – 20	5	20	10
	phyllosilicates	—	—	40 – 50	—	—	60
	thereof:						
	smectite			40			0
	illite/smectite			20			<10
	illite			30			10
	kaolinite			10			10
	sec. chlorite			0			70
		**high-P topsoil**			**high-P subsoil**		
		sand	silt	clay	sand	silt	clay
		(weight-% of total bulk soil mass)					
		7	65	28	18	68	14
		sand	silt	clay	sand	silt	clay
		(weight-% of fraction mass)					
minerals	quartz	30	60	20	20	70	20
	augite	30	30	5	40	20	5
	olivines	10	—	—	30	—	—
	plagioclase	30	—	—	10	—	—
	amphibole	—	minor	—	—	minor	—
	Mg chlorite	—	minor	—	—	minor	—
	mica	—	10	—	—	5	—
	phyllosilicates	—	—	75	—	—	75
	thereof:						
	illite			50			50
	kaolinite			10			10
	sec. chlorite			30			20
	prim. chlorite			10			20

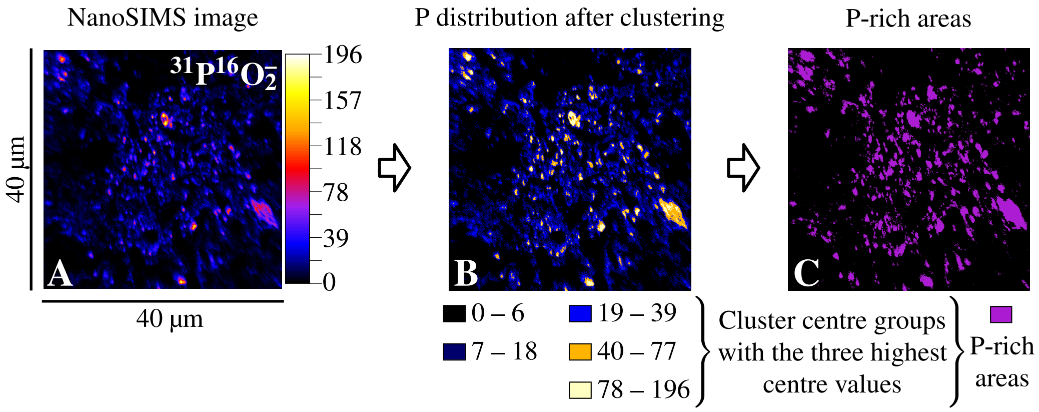

Figure S1. Example of cluster analysis. Nanoscale Secondary Ion Mass spectrometry (NanoSIMS) image of phosphorus (P) from the high-P site subsoil, B) P distribution as clustered image (by k-means clustering, using five cluster centres), C) P-rich areas as defined as areas that are included in the cluster centre groups with the three highest centre values.

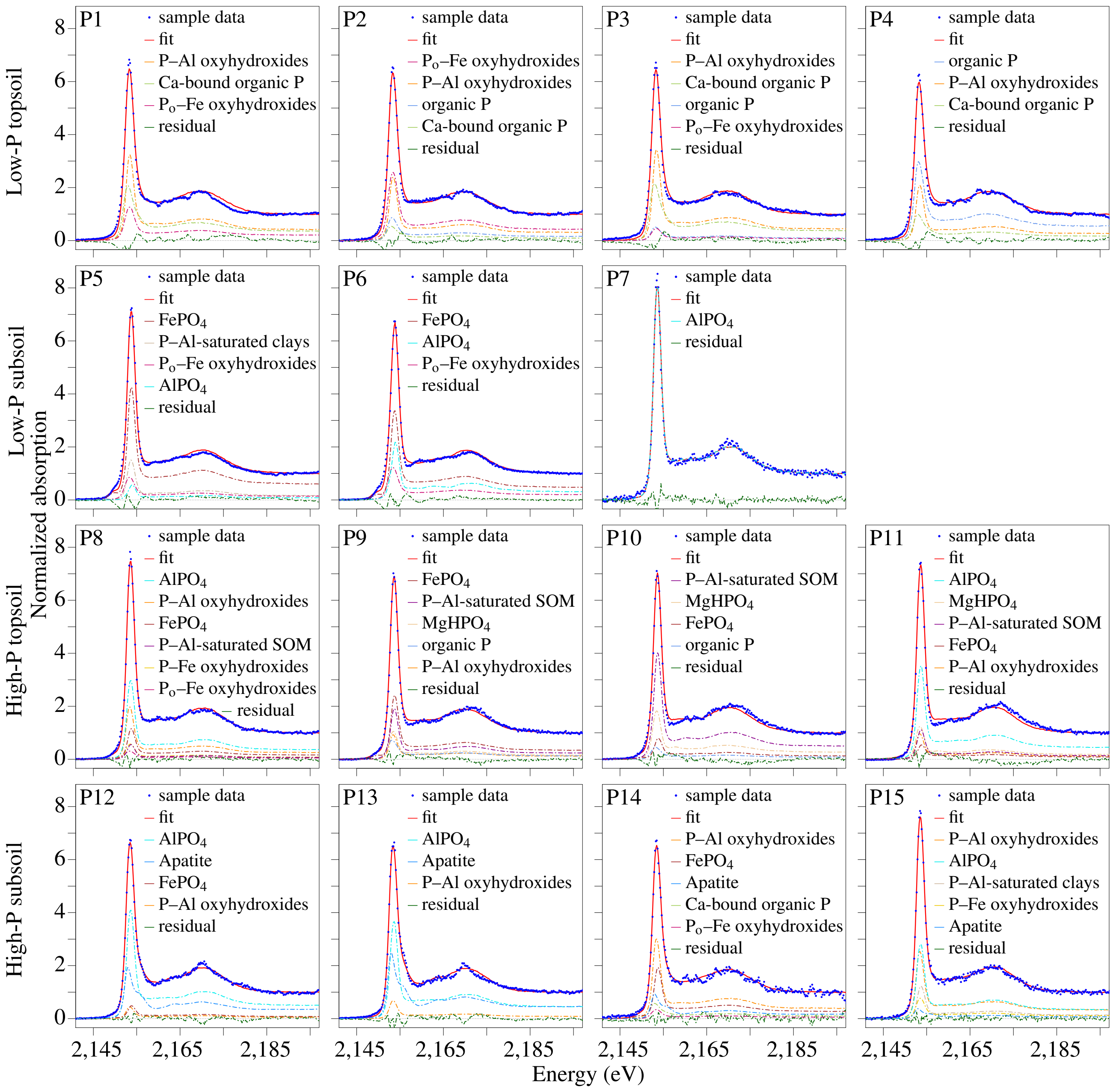

Figure S2. Micro-scale X-Ray Absorption Near Edge Structure (µ-XANES) spectra at the phosphorus (P) *K*-edge. Measurements were conducted at Beamline ID21 of the synchrotron at ESRF, France. Speciation was evaluated at 15 P micro-sites (three or four sites per soil sample), using all relevant P species for linear combination fitting. Also shown are the detected shares of P species and the residual.

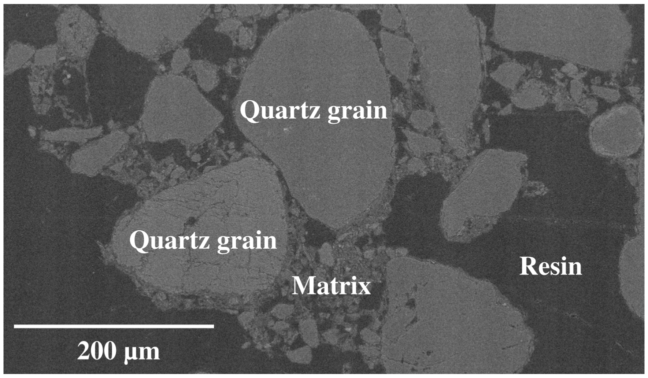

Figure S3. **Scanning electron microscope image of cross-sectioned, quartz-rich aggregate from the low-P subsoil.** Quartz grains (larger particles) are encompassed with and agglutinated by a finer grained matrix. Back scattered electron image.

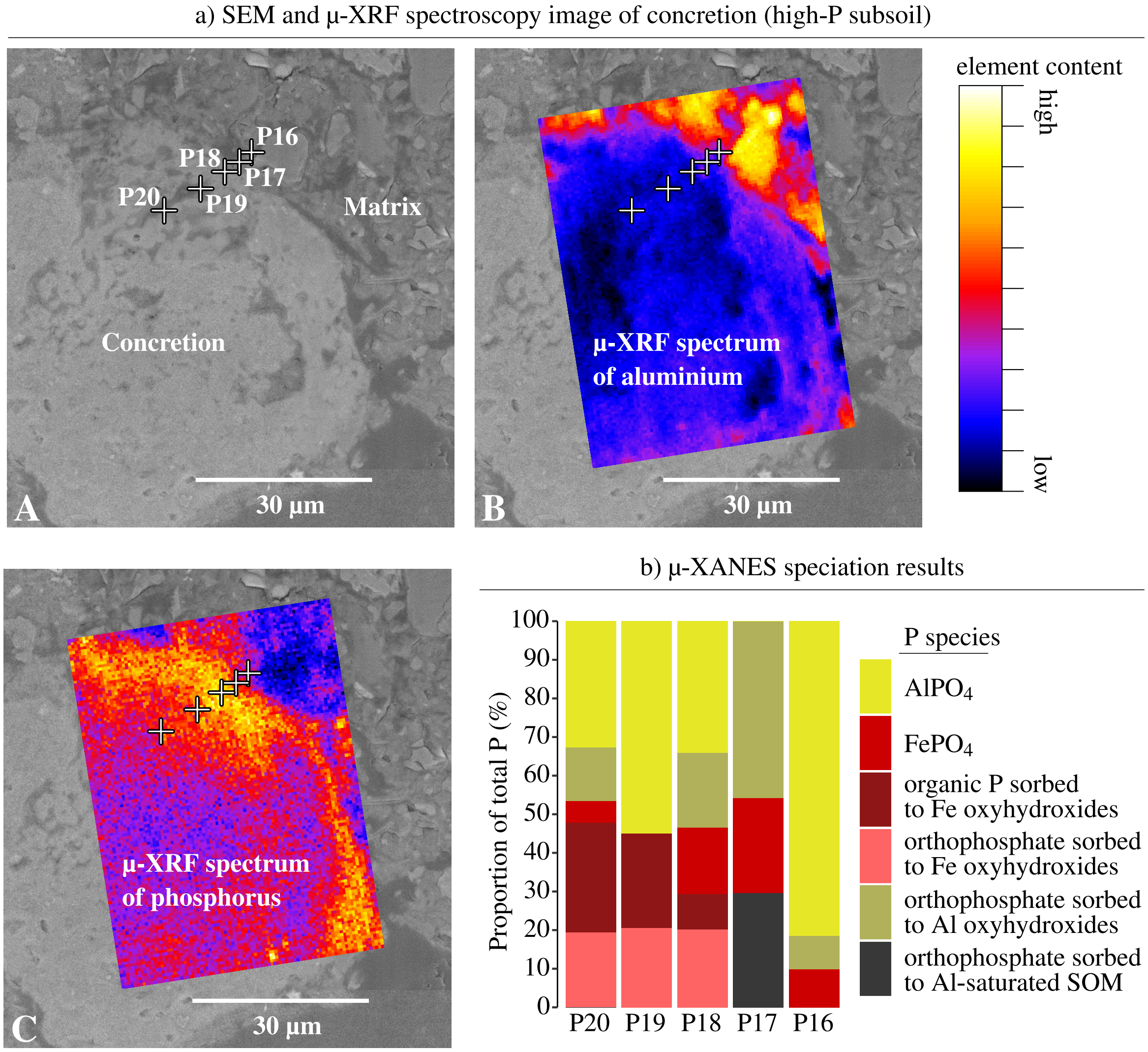

Figure S4. **Transect of micro-scale X-Ray Absorption Near Edge Structure (µ-XANES) speciation from the soil aggregate matrix into an Al-Fe concretion.** a) Scanning electron microscope (SEM) (back scattered electron) images of the concretion and the soil aggregate matrix (A) from the high-Phosphorus (P) subsoil, overlayed by micro-scale X-Ray Fluorescence (µ-XRF) spectroscopy images of aluminium (B) and phosphorus (C), b) transect of five points from the matrix to the inner part of the concretion (P16 $\rightarrow$ P20) was selected for µ-XANES speciation. Proportions of total P are results of linear combination fitting in which only the stated P species were detected in significant amounts. Accuracy of the fitting 5–10 %.

References

1. Saunders, W. M. H. & Williams, E. G. Observations on the Determination of Total Organic Phosphorus in Soils. *J. Soil Sci.* **6**, 254–267 (1955). DOI 10.1111/j.1365-2389.1955.tb00849.x.
2. Mehra, O. P. & Jackson, M. L. Iron Oxide Removal from Soils and Clays by a Dithionite-Citrate System Buffered with Sodium Bicarbonate. *Clays Clay Miner.* **7**, 317–327 (1958). DOI 10.1346/ccmn.1958.0070122.
3. Holmgren, G. G. A Rapid Citrate-Dithionite Extractable Iron Procedure. *Soil Sci. Soc. Am. J.* **31**, 210–211 (1967). DOI 10.2136/sssaj1967.03615995003100020020x.
4. Schwertmann, U. Differenzierung der Eisenoxide des Bodens durch Extraktion mit Ammoniumoxalat-Lösung. *Z. Pflanzenernähr. Düng. Bodenk.* **105**, 194–202 (1964). DOI 10.1002/jpln.3591050303.
